# COVID-19 and multiple crises in Afghanistan: an urgent battle

**DOI:** 10.1186/s13031-021-00406-0

**Published:** 2021-09-17

**Authors:** Mohammad Yasir Essar, Mohammad Mehedi Hasan, Zarmina Islam, Mehr Muhammad Adeel Riaz, Abdullahi Tunde Aborode, Shoaib Ahmad

**Affiliations:** 1 Kabul University of Medical Sciences, Kabul, 1001 Afghanistan; 2 Department of Biochemistry and Molecular Biology, Faculty of Life Science, Mawlana Bhashani Science and Technology University, Tangail, Bangladesh; 3 Dow University of Health Sciences, Karachi, Pakistan; 4 Punjab Medical College, Faisalabad, Pakistan; 5 Healthy Africans Platform, Research and Development, Ibadan, Nigeria; 6 West African Academy of Public Health, Abuja, Nigeria

**Keywords:** COVID-19, Vaccination, Political instability, Healthcare workers, Public health crisis

## Abstract

The political and military advance of the Taliban, reduced healthcare capacity, and imminent humanitarian crisis risk exacerbating an already very serious threat posed by COVID-19 in Afghanistan. The continued rise of COVID-19 cases in Afghanistan appears inevitable, but poor diagnostic capacity prevents accurate case measurement, while vaccine provision is extremely limited. This letter highlights how the recent changes in Afghanistan risk exacerbating the COVID-19 and ongoing health emergency in the country.

## Commentary

COVID-19 is one of many crises facing Afghanistan. Over the last few decades, wars and internal strife have placed Afghanistan’s public infrastructure in jeopardy. Since the announced withdrawal of US and NATO troops from Afghanistan, the country’s situation has become a humanitarian crisis [1]. The Taliban have now gained complete control over the country, but face dealing with mass internal and external displacement of Afghan civilians, monetary inflation and humanitarian concerns [2].

These volatile circumstances are happening in the context of a significant increase in COVID-19 cases as part of a third wave of the pandemic in Afghanistan, particularly from the Delta variant with 60 % of cases reported from Kabul in June from this variant. Now all 34 provinces have declared a COVID-19 situation similar to India’s situation [3]. On June 16, the highest record of 2,313 cases were reported in one day, and in July the country noted a 48 % positivity rate [4]. As of Sept 6 2021, 153,534 COVID-19 positive cases have been reported, of whom 7,141 died [5]. However, the indicated numbers are not accurate due to the country’s low testing capacity with only 664,045 tests administered for a population of 40.4 million, the absence of a national death register, and weak infrastructure with only 35 laboratories across the nation (with some provinces having no laboratories) [4]. Current daily testing capacity is only 4,000 and plans to scale up testing capacity to 8,500 tests a day are now uncertain under the new Taliban regime. The repercussions of this have been drastic for healthcare capacity: bed shortages, lack of oxygen, and low vaccination capacity have raised pressure on hospitals [6, 7]. Despite attempts to increase in the number of beds in Kabul, the capacity rate remains near 100 %. In addition, a reduced number of healthcare workers (HCWs) is another concern; there are only 9.4 HCWs and 1.9 physicians per 10,000 people [8]. Beyond HCWs burnout, lack of training in COVID-19 preparedness and protective equipment is another concern. Due to lack of personal protective equipment, 70 HCWs in Kunduz Regional Hospital tested positive for COVID-19 in 2020 [9]. The United Nations Assistance Mission in Afghanistan (UNAMA) reports attacks on HCWs, which have jumped from 65 to 2018 to 75 incidents in 2019 and are projected to be higher with current conditions [8]. Even prior to the pandemic, hospitals have been a target for political violence, including 140 healthcare facilities that were forcefully closed in 2018 and the attack on a 55-bed government-run maternity hospital in May 2020 [9].

Furthermore, most of the population lives in rural areas, which brings geographical concerns for addressing the pandemic. Healthcare access and security are low in such areas due to lack of provision of general healthcare services [10]. With an unstable political situation, mass migration from rural areas to Kabul, and underlying socioeconomic challenges, providing even minimal health care access has become nearly impossible.

Vaccine coverage remains very low. Vaccine hesitancy is not the primary issue, rather it is adequate supply and administration of vaccines. A survey conducted by Arash et al. shows that 63 % of participants are willing to receive the vaccine [11]. Afghanistan has received 3,068,000 doses of COVID-19 vaccines and was expected to receive 2,192,000 additional doses in the upcoming month, and the US had promised 1,400,000 million doses [4]. However, such efforts have been halted because of Taliban insurgency. These shortcomings, coupled with recent droughts, food shortages, lack of adequate drug supply, unmet funding requirements to fight the pandemic, and black fungus cases, have exacerbated the country’s public health problems [3, 6, 7, 12].

Additionally, the mental health needs will further increase due to the political, humanitarian and COVID-19 crises. An estimated half the population has dealt with depression, anxiety, or post-traumatic stress [13], and inter-generational trauma is likely to further increase future needs. The withdrawal of US and NATO troops has raised many more questions for Afghanistan’s security, particularly for the rights and freedoms of women [14]. However, funding and resources for mental health remain woefully inadequate in Afghanistan [9, 12].

For the betterment of the situation, the following recommendations should be put forth into action. First, humanitarian organizations should be allowed to continue their humanitarian work despite current circumstances. Their work is critical to addressing the recent humanitarian crisis. Negotiations between the international community and the Taliban are necessary to ensure the health and safety of Afghans and humanitarian workers. Second, international allies need to add pressure on the Taliban to ensure human rights are not violated, education continues to be available for both males and females, female healthcare workers should continue their work without any threat, access to health services should be assured, and a national strategy created to control the pandemic and promise the security of Afghan civilians. Third, the international community should continue to support the healthcare system, particularly with vaccine supply, protective personal equipment, testing capacity, medical equipment and monetary support for HCWs.

## Conclusions

At this critical juncture, COVID-19 poses a major risk to the people of Afghanistan in the context of political instability, humanitarian crises and a fragile health system. This creates a complex trajectory for aiding a nation still recovering from the consequences of four decades of war before the pandemic. Moreover, targeting healthcare workers coupled with burnout and mental health concerns makes it difficult to overcome this public health crisis. To this end, Afghanistan needs major international support to control COVID-19.

## Data Availability

Not applicable.

## Abbreviations

COVID-19: Coronavirus disease 2019
HCWs: Healthcare workers
UNAMA: United Nations Assistance Mission in Afghanistan
